# Droplet digital PCR analysis of *CDH13* methylation status in Slovak women with invasive ductal breast cancer

**DOI:** 10.1038/s41598-024-65580-6

**Published:** 2024-06-26

**Authors:** Ivana Baranová, Marek Samec, Dana Dvorská, Igor Šťastný, Katarína Janíková, Ivana Kašubová, Andrea Hornáková, Eva Lukáčová, Andrea Kapinová, Kamil Biringer, Erika Halašová, Zuzana Danková

**Affiliations:** 1 Biomedical Centre Martin, Jessenius Faculty of Medicine in Martin, Comenius University in Bratislava, Martin, Slovakia; 2 Department of Pathological Physiology, Jessenius Faculty of Medicine in Martin, Comenius University in Bratislava, Martin, Slovakia; 3 Biobank for Cancer and Rare Diseases, Jessenius Faculty of Medicine in Martin, Comenius University in Bratislava, Martin, Slovakia; 4 Department of Medical Biology, Jessenius Faculty of Medicine in Martin, Comenius University in Bratislava, Martin, Slovakia; 5 Clinic of Obstetrics and Gynecology, Jessenius Faculty of Medicine in Martin and Martin University Hospital, Comenius University in Bratislava, Martin, Slovakia; 6 Department of Molecular Biology and Genomics, Jessenius Faculty of Medicine in Martin, Comenius University in Bratislava, Martin, Slovakia

**Keywords:** *CDH13*, Droplet digital PCR, CpG methylation, MS-MLPA, Cancer, Molecular biology, Biomarkers

## Abstract

Identifying novel epigenetic biomarkers is a promising way to improve the clinical management of patients with breast cancer. Our study aimed to determine the methylation pattern of 25 tumor suppressor genes (TSG) and select the best methylation biomarker associated with clinicopathological features in the cohort of Slovak patients diagnosed with invasive ductal carcinoma (IDC). Overall, 166 formalin-fixed, paraffin-embedded (FFPE) tissues obtained from patients with IDC were included in the study. The methylation status of the promoter regions of 25 TSG was analyzed using semiquantitative methylation-specific MLPA (MS-MLPA). We identified *CDH13* as the most frequently methylated gene in our cohort of patients. Further analysis by ddPCR confirmed an increased level of methylation in the promoter region of *CDH13*. A significant difference in *CDH13* methylation levels was observed between IDC molecular subtypes LUM A versus HER2 (*P* = 0.0116) and HER2 versus TNBC (*P* = 0.0234). In addition, significantly higher methylation was detected in HER2+ versus HER2- tumors (*P* = 0.0004) and PR− versus PR+ tumors (*P* = 0.0421). Our results provide evidence that alteration in *CDH13* methylation is associated with clinicopathological features in the cohort of Slovak patients with IDC. In addition, using ddPCR as a methylation-sensitive method represents a promising approach characterized by higher precision and technical simplicity to measure the methylation of target CpGs in *CDH13* compared to other conventional methods such as MS-MLPA.

## Introduction

According to the latest official statistics, breast cancer (BC) now represents the most commonly diagnosed malignancy and the leading cause of cancer-related deaths in women worldwide^1,2^. Although mammography, the most widely used technique for BC screening, is an effective tool for early BC detection and significantly reduces the mortality of BC patients, its sensitivity and specificity remain dissatisfactory^3^. Therefore, early BC detection based on more sensitive and specific screening methods is still desirable, which could be reflected in the better prognosis and selection of less severe but effective treatment options^3,4^. Novel molecular biomarkers extensively analyzed by high-throughput omics-based approaches seem to be promising tools not only for improving the diagnosis and prognosis of BC but also for setting up effective targeted personalized therapy in BC patients in the near future.

BC is a very heterogeneous disease at both the molecular and clinical levels, associated with various genetic and epigenetic dysregulations. As epigenetic changes are reversible, they represent an attractive target for BC treatment. DNA methylation is the most extensively studied epigenetic modification that plays a crucial role in the regulation of gene expression. In the human genome, it occurs predominantly on cytosine-phosphate-guanine (CpG) dinucleotides found mostly at the 5´ promoter regions generally called CpG islands (CGIs) of about 70% of genes^5–8^. The DNA methylation of CGIs regulates gene expression through gene-specific repression of transcription^9^. Aberrant promoter methylation of tumor suppressor genes (TSG), which normally inhibits tumor cell progression, invasion, and metastasis, is a frequent and early event in the process of carcinogenesis^10–13^. A wide body of evidence from cancer research has demonstrated that hypermethylation of DNA CGIs, together with hypoacetylated and hypermethylated histones, represent three frequent epigenetic events responsible for the repression of TSG^14–16^. The results from preclinical and clinical BC studies indicate that aberrant DNA methylation patterns of multiple TSG correlate with tumor stage and size, positive lymph nodes, and premenopausal age at diagnosis, and therefore, might be used as a biomarker for diagnosis and therapeutic strategies in BC patients^17–19^. However, as BC is a very heterogeneous disease, methylation status and the type of TSG are inconsistent among different BC studies, and the proper frequency and utility of DNA methylation as a biomarker in BC patients has not yet been established. Further studies in this area are certainly necessary.

Aberrantly methylated genes in promoter regions of TSG represent frequent events in BC carcinogenesis^20^. Recent genome-wide studies revealed abnormal methylation state of key genes participating in the occurrence, development, and maintenance of cancer^21^. Accelerating the development of approaches based on sequencing or array technologies improved DNA methylation profiling, which allows a deeper understanding of the biological processes behind the methylation landscape^22^. On the other hand, these methods for DNA methylation profiling require expensive reagents, labor time, and bioinformatics analysis to summarize a large amount of data; thus, it is unlikely that these technologies will be implemented into routine clinical practice in the near future^23^. Semiquantitative methods such as MS-MLPA can overcome shortcomings associated with genome-wide DNA methylation analysis, mainly in cost and bioinformatics processing. MS-MLPA allows the simultaneous analysis of the DNA methylation state in several genes, but this technique is limited by its dependence on the presence of the *Hha*I restriction site^24^. Despite the mentioned limitations, MS-MLPA can be used as a laboratory assay for robust, reliable, and semiquantitative detection of DNA methylation in promoter regions of several TSGs.

The detection of methylation state of DNA in a single gene is a rapidly advancing field of molecular medicine, offering the potential to distinguish between different tumor types and predict their response to chemotherapeutic intervention regarding their specific methylation profile. Currently, several methods are available to determine the methylation status of DNA samples based on bisulfite modification, biological identification, or chemical cutting^25^. Selecting an appropriate method for determining the methylation profile of specific promoter regions of individual TSG with high specificity, sensitivity, and cost-effectiveness is critical for acquiring significant experimental data and subsequent clinical application^26^. Droplet digital PCR (ddPCR) is a relatively novel method that enables the precise detection and quantification of target DNA. This method allows distinguished changes in DNA with single-base resolution, and therefore, it represents a suitable approach to detecting differently methylated CpG in the promoter region of TSG^27^. Moreover, detecting methylated DNA derived from primary tumors is currently a challenge and requires a highly sensitive and specific assay^28^.

Based on the above-mentioned knowledge, the main aim of our research work was to identify the novel methylation biomarkers in tissue samples of Slovak patients with invasive ductal carcinoma (IDC) that would correlate with their clinicopathological characteristics.

## Materials and methods

The study group consisted of 166 women diagnosed with IDC. The clinicopathological characteristics of the patients are summarized in Table 1. Histologically normal tissue adjacent to tumor from 10 patients was used as control samples. Formalin-fixed, paraffin-embedded (FFPE) tissue samples were provided by the Department of Pathological Anatomy, Jessenius Faculty of Medicine in Martin, Comenius University and University Hospital in Martin. The study was designed in line with the ethical principles of the Helsinki Declaration and approved by the Ethics Committee of Jessenius Faculty of Medicine (EK1822/2016). All the patients enrolled in the study signed informed consent.

**Table 1:** Clinicopathological characteristics of patients enrolled in the study (n = 166).

Age	< 55	n = 53	31.9%
	≥ 55	n = 113	68.1%
Grade	1	n = 24	14.5%
	2	n = 37	22.3%
	3	n = 105	63.3%
Tumor status	pT1	n = 84	50.6%
	pT2	n = 63	38.0%
	pT3	n = 7	4.2%
	pT4	n = 10	6.0%
	pTx	n = 2	1.2%
Nodal status	pN0	n = 89	53.6%
	pN1	n = 32	19.3%
	pN2	n = 19	11.4%
	pN3	n = 14	8.4%
	pNx	n = 12	7.2%
Molecular classification	HER2	n = 40	24.1%
	LUM A	n = 66	39.8%
	LUM B	n = 14	8.4%
	TNBC	n = 46	27.7%
ER status	ER+	n = 79	47.6%
	ER-	n = 87	52.4%
PR status	PR+	n = 70	42.2%
	PR-	n = 96	57.8%
HER2 status	HER2+	n = 54	32.5%
	HER2-	n = 112	67.5%

ER, estrogen receptor; PR, progesterone receptor; LUM, luminal; TNBC, triple-negative breast cancer.

### DNA isolation and bisulfite modification

FFPE tissue samples were deparaffinized with xylene. The DNeasy Blood and Tissue kit (Qiagen, Hilden, Germany) was then used for DNA isolation. The obtained DNA concentration was measured with a fluorometer Qubit 3.0 (Thermo Fisher Scientific, Waltham, MA, USA) using a dsDNA BR Assay kit (Thermo Fisher Scientific). For methylation-specific MLPA (MS-MLPA) analysis, the isolated DNA was adjusted to 15 ng/µl and stored at -20 °C. For further methylation analysis utilizing droplet digital PCR (ddPCR), one µg of isolated DNA was modified with an EpiTect Bisulfite kit (Qiagen, Hilden, Germany) following the manufacturer´s instructions.

### Methylation-specific MLPA

MS-MLPA analysis was employed to detect methylation levels of 25 tumor suppressor genes in SALSA MLPA ME002 Tumour suppressor mix2 (MRC Holland, Amsterdam, The Netherlands). Restriction endonuclease *HhaI* (Promega, Southampton, UK) was used to cleave the samples. The probe mix was composed of 41 probes, 27 containing the cleavage sites for methylation detection. Samples were prepared and analyzed according to the kit manufacturer's instructions. The individual reaction steps were carried out on Sure Cycler 8800 thermocycler (Agilent Technologies, Santa Clara, CA, USA). PCR amplifications were followed by fragment analysis on the ABI 3500 genetic analyzer (Thermo Fisher Scientific). The results were analyzed using Coffalyser.Net software (MRC Holland). The individual baseline methylation level was calculated for each methylation-specific probe as suggested by the manufacturer, by taking the mean methylation of control samples per probe and adding two times the standard deviation. The calculated value was considered the cut-off for determining the methylation status of each sample.

### Methylation analysis of *CDH13* gene

Increased methylation in the *CDH13* gene detected by MS-MLPA was further investigated using ddPCR analysis. Primers and probes were designed using the MethPrimer^31^ and Primer3Plus^32^ online programs. However, it was unfeasible to achieve precise alignment with the specific CpG site detected by the MS-MLPA probe (chr16:82,627,075 in hg38 assembly). Instead, the designed probes targeted three nearby CpG sites (chr16:82,626,843; chr16:82,626,845; chr16:82,626,859) in the *CDH13* promotor region. The similarity of methylation patterns between the CpG site detected by the MS-MLPA probe and the CpG sites targeted by our probes was evaluated using The Cancer Genome Atlas data. The methylation array in the Breast Cancer project covered two of our CpG sites (chr16:82,626,845 and chr16:82,626,859) and a CpG site 10 bp downstream from the MS-MLPA probe (hg38; chr16:82,627,065). The methylation pattern of those CpG sites in 295 IDC samples was very similar. The designed primers and probes sequences are presented in Table 2.

**Table 2:** Primers and probe sequences for *CDH13* methylation detection by droplet digital PCR.

Primer/Probe	Primer/Probe Sequence 5´ → 3´
Forward Primer	AAAGAAGTAAATGGGATGTTATTTTC
Reverse Primer	ACCAAAACCAATAACTTTACAAAAC
M-Probe (FAM)	TCGCGAGGTGTTTATTTCGT
UnM-Probe (HEX)	TTTTGTGAGGTGTTTATTTTGTATTTGT

M, methylated; UnM, unmethylated.

The ddPCR assay was optimized for simultaneous detection of methylated and unmethylated sequences using a probe targeting the methylated DNA (M-Probe, FAM labeled) and a probe targeting the unmethylated DNA (UnM-Probe, HEX labeled) in one reaction mix. Duplex ddPCR mixture contained 10 μL of Supermix for Probes (No dUTP) (Bio-Rad Laboratories, Hercules, CA, USA), 0.45 μL of each primer, 0.45 μL of each probe, 2.5 μL of bisulfite converted DNA adjusted with 6.1 μL of water up to final volume of 20 μL. For control purposes, commercially available fully methylated and unmethylated EpiTect DNA controls (Qiagen, Hilden, Germany) were used, along with ultrapure water, to ascertain the absence of reagents contamination. The entire reaction volume, along with 70 μL of Droplet Generation Oil for Probes, was loaded into a DG8 cartridge and inserted into the QX200 Droplet Generator (Bio-Rad Laboratories) to generate approximately 20,000 droplets per sample. The resulting droplet emulsion (40 μL) was transferred into a 96-well PCR plate, covered with a pierceable foil, and heat-sealed using Bio-Rad's PX1 system. Subsequent PCRs were carried out in the T100 thermal cycler (Bio-Rad Laboratories). Optimized PCR protocol was initialized with denaturation (95 °C for 10 min), followed by 40 cycles of denaturation (94 °C for 30 s) and annealing/extension (57 °C during for 1 min) with ramp rate decreased to 1 °C/s. The enzyme was deactivated at 98 °C for 10 min, and the product was held at 12 °C overnight according to the recommendations by Rowlands et al.^33^. Following the amplification step, the plate was loaded onto the QX200 Droplet Reader (Bio-Rad Laboratories) for droplet analysis of each individual well.

**Figure 1:**
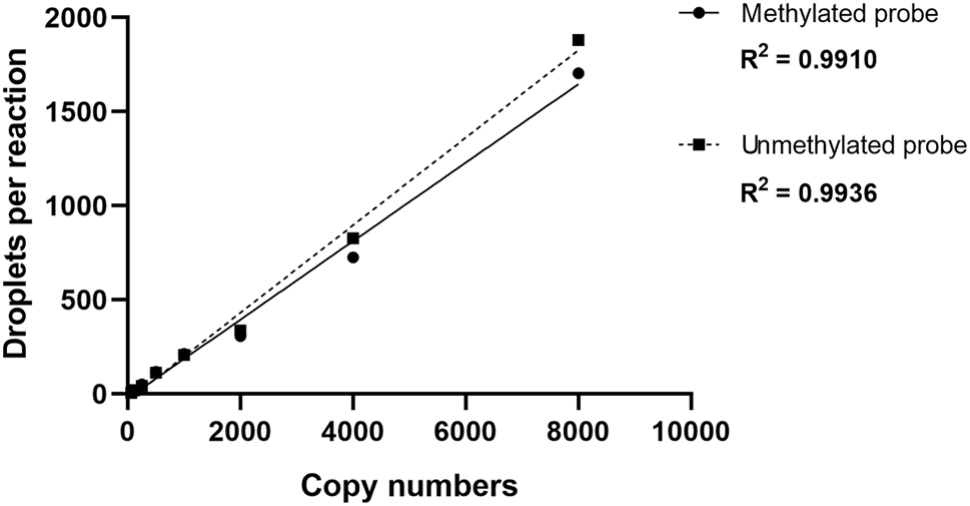
The linearity of ddPCR assay for quantification of *CDH13* methylation.

The QuantaSoft v.1.7 Software (Bio-Rad Laboratories) was utilized to analyze and categorize each droplet based on fluorescence emission detected in HEX or FAM channels. The droplets were sorted into four clusters 1) methylated DNA with FAM signal, 2) unmethylated DNA with HEX signal, 3) DNA with both signals, and 4) empty droplets lacking the target DNA. The threshold amplitudes were determined for FAM and HEX signals at values 2600 and 1745, respectively. The correlation coefficient (R^2^) was calculated by performing serial dilutions of methylated and unmethylated EpiTect DNA controls in water, with 8000, 4000, 2000, 1000, 500, 250, 125, and 62 copies per reaction (Fig. 1). For each dilution specificity and sensitivity was calculated: specificity as copies of the unmethylated control DNA detected by the UnM-Probe divided by all copies of the unmethylated control DNA (detected by both probes); sensitivity as the methylated control DNA detected with the M-Probe divided by all copies of the methylated control DNA. As no copies of unmethylated control DNA were detected by the M-Probe, the specificity reached 100%. The average sensitivity across all dilutions was 96.5%.

The level of methylation was expressed as a percentage, representing the ratio of methylated sequences to the sum of methylated and unmethylated sequences.

### Statistical analysis

Statistical analysis was performed for 135 cases with available clinicopathological data that underwent analysis by ddPCR. The Shapiro–Wilk test was used to check the normality assumption. The association between *CDH13* methylation levels and clinicopathological features, such as ER, PR, and HER2 status, was analyzed using a Mann–Whitney test. The Kruskal–Wallis one-way ANOVA was used to evaluate the association between *CDH13* methylation levels and grade, tumor status, nodal status, molecular classification, and age. Associations between clinicopathologic features and the number of samples with detected methylation (cut-off 10%) were analyzed using Fisher´s exact test. A value of *P* < 0.05 was considered as statistically significant. All statistical analyses were performed in GraphPad Prism 8.1.1 (GraphPad, San Diego, CA, USA). Additionally, a heatmap was constructed to visualize the results obtained from MS-MLPA analysis.

## Results

**Figure 2:**
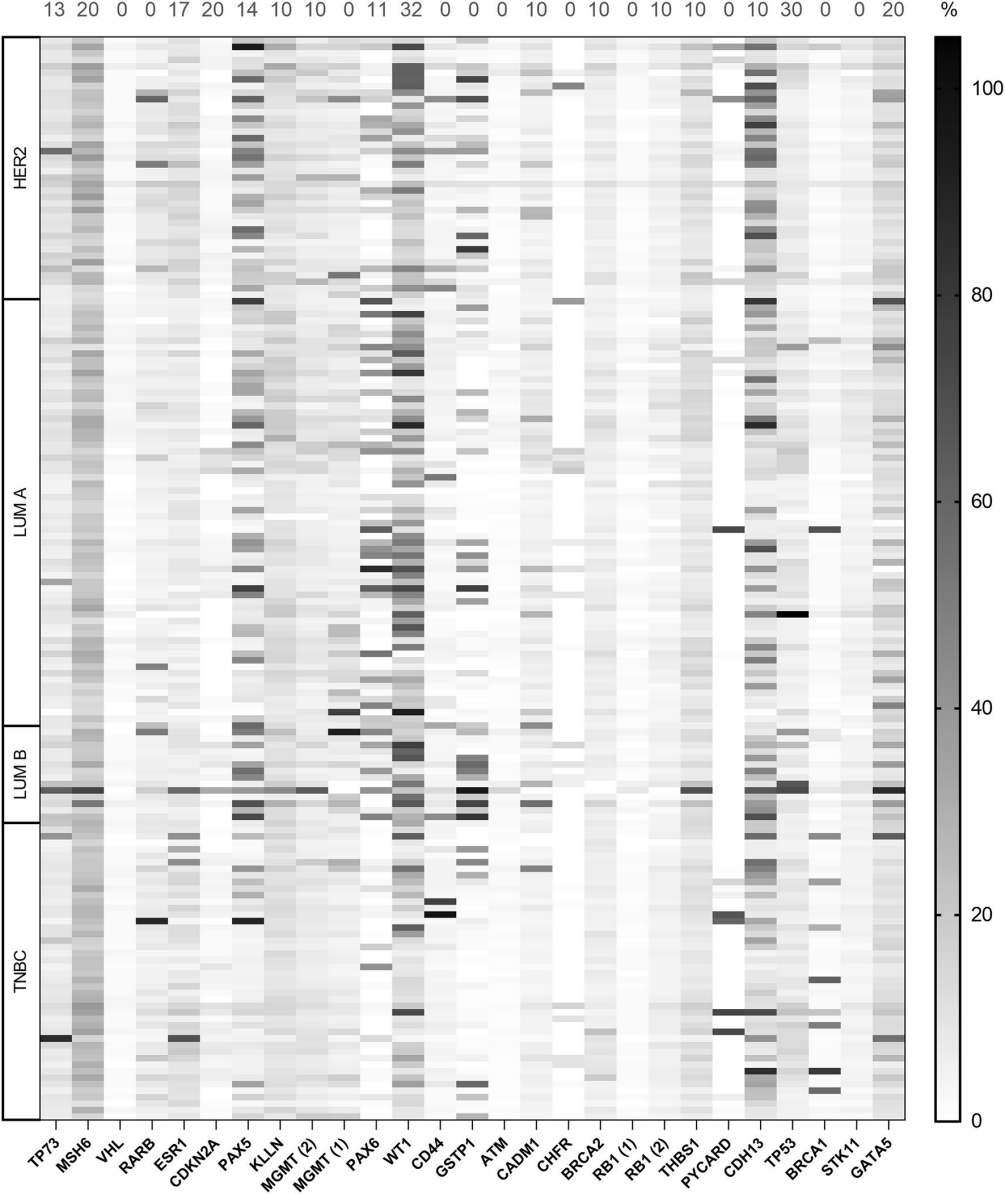
Heatmap visualization of methylation levels detected by MS-MLPA. The cut-off value discriminating between methylated and unmethylated status for each gene is shown at the top of the heatmap. LUM, luminal; TNBC, triple-negative breast cancer.

In the first part of our study, we performed MS-MLPA analysis to obtain an overview of changes in promotor methylation of 25 tumor suppressor genes. Using calculated baseline methylation level for each tumor suppressor gene, we observed an increase of methylation in 13 genes (*TP73*, *MSH6*, *RARB*, *PAX5*, *KLLN*, *MGMT*, *PAX6*, *WT1*, *GSTP1*, *CADM1*, *THBS1*, *CDH13*, and *GATA5*). Significantly increased methylation was most often present in *CDH13* (67.5% of analyzed samples), *MSH6* (65.1%), *KLLN* (61.5%), *PAX5* (44.0%), *THBS1* (43.4%), and *WT1* (31.9%). The rest of the examined genes (*VHL*, *ESR1*, *CDKN2A*, *CD44*, *ATM*, *CHFR*, *BRCA2*, *RB1*, *PYCARD*, *TP53*, *BRCA1* and *GATA5*) did not exhibit any change in methylation or methylation was increased in less than 10% of analyzed samples. The methylation levels of all 25 tumor suppressor genes detected by MS-MLPA are depicted in Fig. 2. We selected *CDH13* as the most frequently methylated gene for further analysis and result validation in the next part of our study.

**Figure 3:**
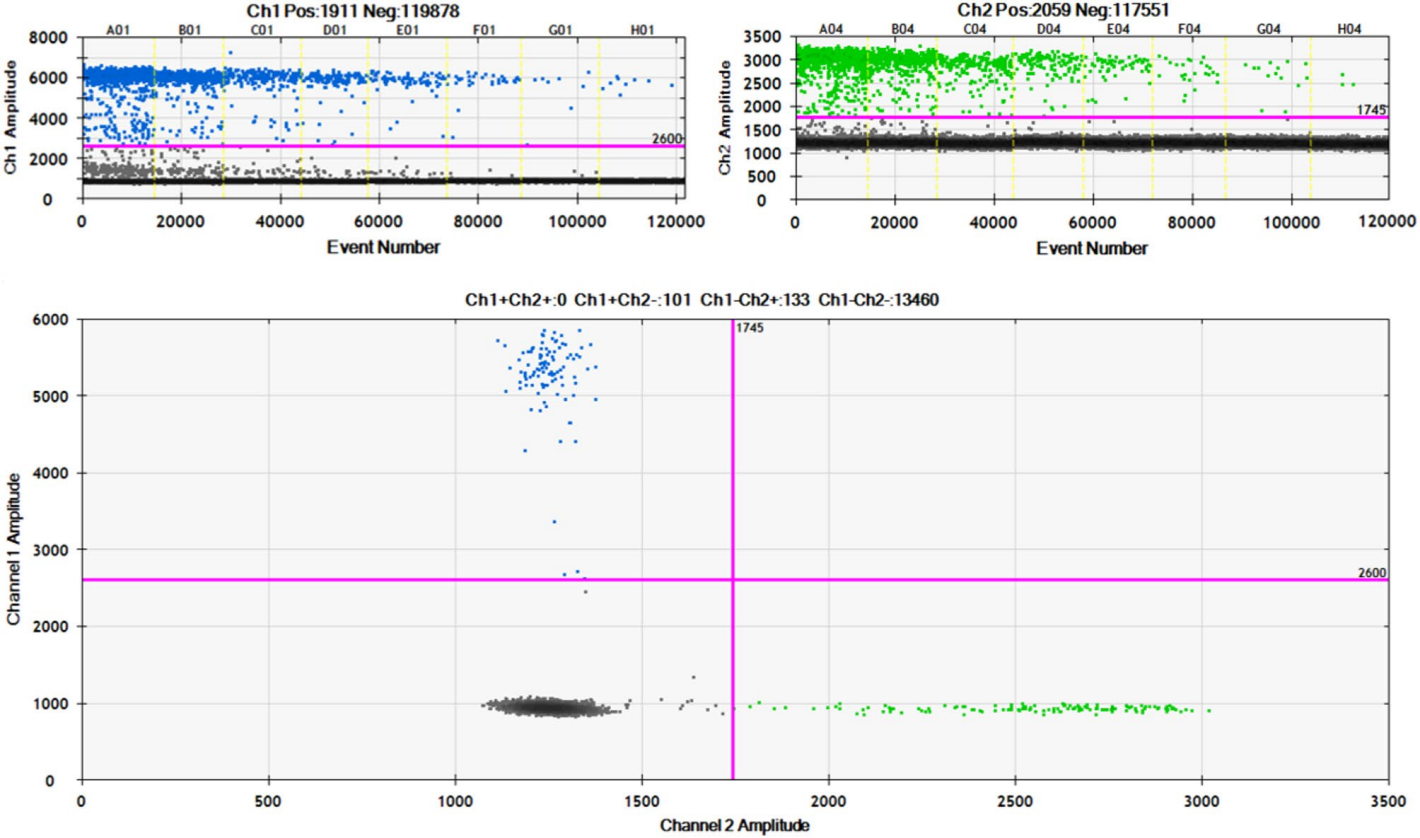
Representative plots of ddPCR results. Two-fold dilution series, diluted from 8000 to 62 copies per reaction, of methylated (top left) and unmethylated control DNA (top right). Two-dimensional plot corresponding to methylated sample (bottom). Blue dots represent a number of DNA copies considered methylated; green dots represent unmethylated copies of DNA. The resulting level of sample methylation is calculated as the ratio between methylated copies and all DNA copies detected by both channels. The purple lines define the threshold amplitudes.

**Figure 4:**
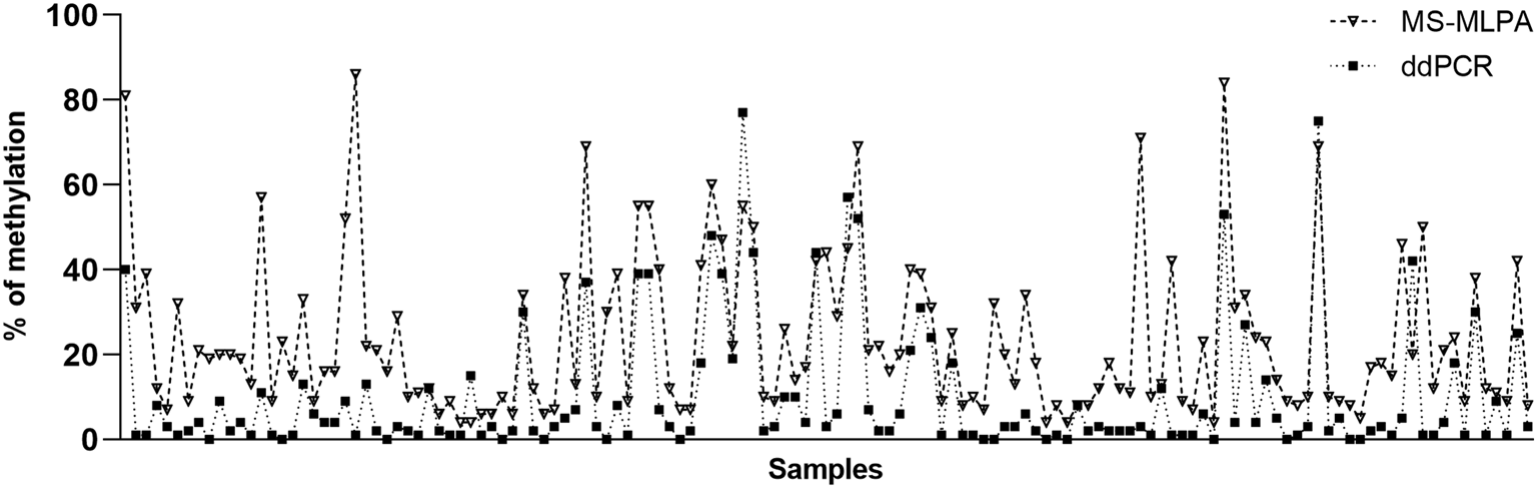
Methylation levels detected by MS-MLPA and ddPCR in *CDH13* gene.

Using ddPCR, we successfully analyzed 135 samples. We were unable to analyze the whole set of 166 samples due to the insufficient amount of some tissue samples or the necessity to exclude the bisulfite-converted DNA of inadequate quality. The sample was considered methylated if methylation level exceeded 10%. We detected methylation in 25.2% of BC samples with an average methylation of level 31.0%. An example of data output from ddPCR analysis is presented in Fig. 3. In the same sample set, MS-MLPA showed methylation in 67.4% of tumor samples with average methylation level 30.9%. The comparison of methylation levels detected in individual samples is depicted in Fig. 4.

**Figure 5:**
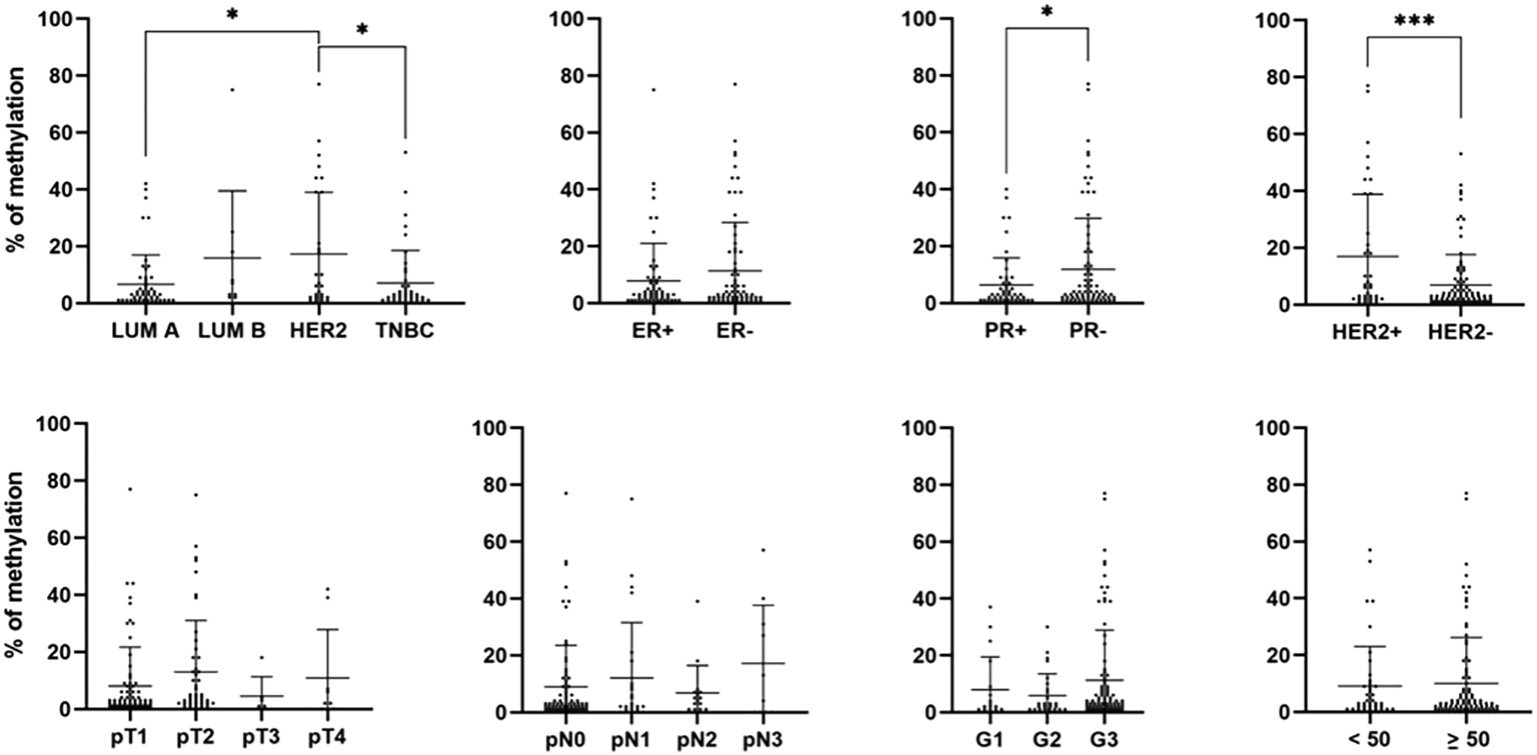
Correlation of detected *CDH13* methylation levels with clinicopathological characteristics. ER, estrogen receptor; PR, progesterone receptor; LUM, luminal; TNBC, triple-negative breast cancer.

In subsequent statistical analysis, we investigated the correlation between clinicopathological characteristics and methylation detected by ddPCR in the promoter region of the *CDH13* gene. Observed methylation was evaluated as methylation levels of all individual samples as well as a number of samples with detected methylation using the cut-off of 10%. A significant difference in *CDH13* methylation was observed between BC molecular subtypes LUM A vs. HER2 (average methylation level 6.54% vs. 17.3%, *P* = 0.0116; 17.0% of methylated samples vs. 43.3%, *P* = 0.0183) and HER2 vs. TNBC (average methylation level 17.3% vs. 7.09%, *P* = 0.0234). Although, the percentage of samples with detected methylation in the HER2 subgroup was 43.3. % vs. 20.9% in the TNBC subgroup, the difference was not considered statistically significant (*P* = 0.0683). The statistically significant differences were also revealed when tumors were compared according to their ER, PR, and HER2 status. Significantly higher methylation was detected in HER2-positive vs. HER2-negative tumors (17.0% vs. 6.79%, *P* = 0.0004; 41.0% vs. 18.8%, *P* = 0.0092) and PR-negative vs. PR-positive tumors (11.9% vs. 6.29%, *P* = 0.0421; 31.3% vs. 15.4%, *P* = 0.0431). Although higher methylation was observed in grade 3 tumors, the difference was not considered statistically significant. A significant difference was also observed between the numbers of methylated samples with pT1 and pT2 tumor status (18.3% vs. 37.5%, *P* = 0.0322); however, the difference between the average methylation levels did not prove statistically significant (8.07% vs. 12.9%, *P* = 0.3390). No significant correlation was observed between *CDH13* methylation and the rest of the recorded clinicopathological characteristics (, nodal status, age). Differences between *CDH13* methylation levels in individual clinicopathological subgroups are presented in Fig. 5. Table 3 summarizes differences according to the number of methylated samples in each group.

**Table 3:** Clinicopathological characteristics versus number of patients with detected *CDH13* methylation.

Characteristic		n (%)	*CDH13* methylation	*P*-value < 0.05
			n (%)	
Age	< 55	43 (31.9%)	10 (7.4%)	
	≥ 55	92 (68.1%)	24 (17.8%)	
Grade	1	18 (13.3%)	4 (22.2%)	
	2	27 (20.0%)	6 (22.2%)	
	3	90 (66.7%)	24 (26.7%)	
Tumor status	pT1	71 (52.6%)	13 (18.3%)	0.0322
	pT2	48 (35.6%)	18 (37.5%)	
	pT3	6 (4.4%)	1 (16.7%)	
	pT4	9 (6.7%)	2 (22.2%)	
	pTx	1 (0.7%)	0 (0.0%)	
Nodal status	pN0	74 (54.8%)	18 (24.3%)	
	pN1	25 (18.5%)	7 (28.0%)	
	pN2	16 (11.9%)	2 (12.5%)	
	pN3	10 (7.4%)	5 (50.0%)	
	pNx	10 (7.4%)	2 (20.0%)	
Molecular classification	HER2	30 (22.2%)	13 (43.3%)	0.0183
	LUM A	53 (39.3%)	9 (17.0%)	
	LUM B	9 (6.7%)	3 (33.3%)	
	TNBC	43 (31.9%)	9 (20.9%)	
ER status	ER+	61 (45.2%)	11 (18.0%)	
	ER−	74 (54.8%)	23 (31.1%)	
PR status	PR+	52 (38.5%)	8 (15.4%)	0.0431
	PR−	83 (61.5%)	26 (31.3%)	
HER2 status	HER2+	39 (28.9%)	16 (41.0%)	0.0092
	HER2−	96 (71.1%)	18 (18.8%)	

ER, estrogen receptor; PR, progesterone receptor; LUM, luminal; TNBC, triple-negative breast cancer.

## Discussion

BC is a heterogeneous disease that develops due to the accumulation of genetic and epigenetic changes in cells^34^. DNA methylation, as one of the epigenetic mechanisms, is strongly associated with malignant transformation. Biologically, the specific methylation pattern in individual genes can act as an initial event of carcinogenesis or play an essential role in the promotion and progression of BC^35^. Methylation of the CpG islands of the gene promoter regions is a highly dynamic process linked to gene expression and regulation that potentially fluctuates over time^36^.

In the current study, we determined the methylation status of the promoter region of 25 TSGs, focusing on *CDH13* and its association with clinicopathological characteristics in the cohort of Slovak patients with IDC. To evaluate the hyper-methylation of *CDH13*, we used semiquantitative MS-MLPA as a prescreening method and quantitative ddPCR to confirm the obtained results. Here, we move toward the possible clinical application of *CDH13* promoter methylation by 1) determining the appropriate methods for methylation detection and 2) evaluating clinical association with clinicopathological features of BC to determine the impact of methylation on clinical outcome.

The choice of appropriate high throughput methods for DNA methylation assessment with single-base resolution plays an essential step in identifying methylation patterns associated with clinical outcomes. In the present study, we used two diagnostic approaches to detect differently methylated CpGs in the promoter region of *CDH13*. The MS-MLPA techniques using methylation-sensitive digestion represent an appropriate molecular approach to identify methylation changes in multiple TSG, providing semiquantitative data without bisulfite conversion required for other detection methods such as methylation-specific PCR or quantitative methylation-specific PCR^26^. On the contrary, there are some limitations of MS-MLPA. Each probe can recognize only one specific CpG site; thus, the methylation of another or nearby CpG island cannot be included. To improve the accuracy of target-DNA detection and measurement, we used methylation-specific ddPCR. We developed and optimized a unique ddPCR assay for *CDH13* to evaluate the methylation status of the gene promoter region. This approach overcomes the limitation of MS-MLPA and enables the absolute quantification of methylated DNA in FFPE samples^37^.

MS-MLPA analysis has been used for over a decade to detect epigenetic alterations in the genes associated with cancer development^38^. In the present study, we used an MS-MLPA probe set to evaluate the methylation status of TSG in patients with IDC. We identified an increased methylation frequency in 6 genes, including *MSH6*, *PAX5*, *WT1*, *KLLN, THBS6*, and *CDH13*, in the BC group compared to the control samples. It has been shown that methylation of these genes may serve as a potential marker for the diagnosis and prognosis of BC^39–41^*. Marzese *et al*.* documented frequent *WT1* promoter methylation in a cohort of patients with IDC^42^. Furthermore, in a retrospective study by *Moelans *et al., the authors confirmed a higher *MSH6, CDH13, WT1,* and *PAX5* methylation rate in archival samples obtained from BC patients^43^.

Results acquired from MS-MLPA revealed the most prevalent methylation in the *CDH13* gene. *CDH13* (also known as T-cadherin or H-cadherin)*,* localized to chromosome 16q24, is a unique transmembrane glycoprotein belonging to the cadherin superfamily, characterized by being devoid of a transmembrane domain^40^. Cadherins, including *CDH13*, play an essential role in cell–cell adhesion, and inactivation of this cadherin-mediated cell adhesion due to cadherin downregulation is associated with cancer progression^44^*.* Abnormal *CDH13* methylation is frequently observed in different types of tumors, including BC^45–48^. Re-expression of *CDH13* is associated with the suppression of invasiveness, tumor growth, and neovascularization in BC *in vitro*^40^. For this reason, effective screening and early, accurate detection of aberrantly methylated *CDH13* promoter region could be used as a promising diagnostic and prognostic marker for various malignancies, including breast cancer. To verify the correct performance of MS-MLPA analysis, we developed an in-house methylation-specific ddPCR assay for *CDH13*. We confirmed increased levels of CpG methylation in the target site of the promoter region in tumor samples. As expected, *CDH13* methylation was absent in tumor-adjacent (morphologically normal) tissue, and none of our control samples showed any methylation (cut-off 10%).

Subsequently, we investigated the association between *CDH13* methylation levels and molecular classification and clinicopathological characteristics of BC. We detected higher methylation levels in tumors classified as HER2 compared to LUM A (*P* = 0.0116) and TNBC (*P* = 0.0234). These data are consistent with a previously published study, stating that *CDH13* methylation is significantly higher in HER2 subtypes than in LUM A and TNBC^49^. Our study showed that higher methylated *CDH13* was associated with immunohistochemically verified HER2-positive status (*P* = 0.0004). These data are in agreement with previous studies, which reported increased methylation of *CDH13* in a cohort of HER2-positive BC patients^50^*.* Another study by *Pang *et al*.* identified *CDH13* gene hypermethylation associated with HER2-amplification among patients with ductal carcinoma in situ (DCIS)^51^. Taken together, our results indicate that *CDH13* methylation may play a significant role in different BC phenotypes.

Furthermore, methylation-sensitive ddPCR analysis revealed higher *CDH13* methylation levels in patients with PR-negative status than those with PR-positive tumors (*P* = 0.0421). Consistent with existing literature, our findings corroborated the link between increased *CDH13* methylation and PR-positive status^51,52^. In general, PR-negative status has been shown to have a worse prognosis and survival outcomes compared to PR-positive patients with BC^53^.

In conclusion, we showed that increased *CDH13* methylation is significantly associated with the aggressive phenotype of BC with the potential to serve as a diagnostic and prognostic biomarker. Identifying a specific methylation pattern in the DNA promoter region of targeted genes could enhance the ability to distinguish BC molecular subtypes when combined with routine immunohistochemistry staining, thus enhancing specificity and sensitivity in distinguishing aggressive from indolent BC. *CDH13* serves as an important tumor suppressor responsible for cell–cell adhesion, often deactivated by abnormal promoter hypermethylation during carcinogenesis. Therefore, selecting this cell adhesion-related gene to assess breast cancer prognosis is a logical approach for future clinical implementation.

Precise and accurate measurements of breast cancer-associated biomarkers require developing novel diagnostic methods based on analysis of DNA methylation status. Using ddPCR represents a promising approach for confirming data obtained from semiquantitative analysis such as MS-MLPA, and thus, it could be helpful in the management of Slovak patients with BC.

## Data Availability

All data analyzed during this study are included in this published article. The generated datasets are available from the corresponding author upon reasonable request.
